# MF management

**DOI:** 10.1097/HS9.0000000000000210

**Published:** 2019-06-30

**Authors:** Prithviraj Bose, Srdan Verstovsek

**Affiliations:** Department of Leukemia, University of Texas MD Anderson Cancer Center, Houston, United States

**Take home messages**

- Current medical management of myelofibrosis revolves around the use of ruxolitinib, which is effective regardless of driver mutation status, and spleen responses to which are dose-dependent and correlate with survival. Combination approaches, especially with agents that can ameliorate bone marrow fibrosis and/or counteract ruxolitinib-induced cytopenias are attractive areas of clinical investigation.
- Cytopenias in myelofibrosis remain challenging, and novel approaches are needed. Current studies suggest promise of the activin receptor ligand traps for anemia, and low dose thalidomide (50 mg/d) for thrombocytopenia. Anemia is not a contraindication to ruxolitinib use. Effective treatment for cytopenias remains an unmet need in myelofibrosis.
- Patients that fail ruxolitinib have a dismal prognosis, but ruxolitinib failure remains difficult to define. Many drugs with diverse mechanisms of action, as well as other JAK inhibitors, are being tested in patients with suboptimal response or resistance to ruxolitinib.

## Introduction

Patients with myelofibrosis can present with one or more of the following: cytopenias, most frequently anemia, splenomegaly, constitutional and other symptoms, thrombosis, hemorrhage, extramedullary hematopoiesis, pulmonary hypertension, etc. In our practice, we adopt a clinical needs-oriented approach to management. Despite the recent emergence of many different prognostic models for patients with primary myelofibrosis (PMF), for simplicity and ease of use, we employ the Dynamic International Prognostic Scoring System (DIPSS), but also take into account other well-established adverse clinical and genomic risk factors, such as triple negativity, “high molecular risk” nondriver mutations, for example, *ASXL1*, *SRSF2*, *U2AF1*,^1^ elevated bone marrow blasts, red cell transfusion dependence, unfavorable and “very high risk” karyotypes^2^ and thrombocytopenia when making a decision to refer the patient for allogeneic hematopoietic cell transplantation (allo-HCT). In general, we refer most patients who have DIPSS intermediate-2 or high risk disease for transplant consultation, but also consider it in selected patients with DIPSS intermediate-1 disease who have one or more of the other adverse risk factors mentioned above. For patients with postpolycythemia vera or postessential thrombocythemia myelofibrosis (post-PV/ET MF), who have a more indolent clinical course and in whom traditional models for prognostication derived from studying patients with PMF may not work as well, we use the myelofibrosis secondary to PV/ET prognostic model (MYSEC-PM),^3▪^ which was derived based on a large cohort of patients with post-PV/ET MF.

## Current state of the art

Contemporary drug therapy of myelofibrosis centers on the use of the Janus kinase 1/2 (JAK1/2) inhibitor, ruxolitinib. Importantly, with the exception of *JAK2* V617F allele burden (higher efficacy when ≥50%)^4^ and number of nondriver mutations on multigene profiling by next-generation sequencing (≥3 mutations = lower odds of spleen response and inferior survival),^5^ no factors have been identified that may predict the likelihood, quality, or duration of response to ruxolitinib. As such, we decide on the use of ruxolitinib entirely based on clinical factors. Long-term follow-up of the pivotal COMFORT trials has demonstrated a clear survival advantage of ruxolitinib treatment in patients with IPSS intermediate-2 or high risk myelofibrosis.^6▪^^,^^7▪^ Consistent with Italian consensus guidelines,^8^ however, we do not advocate the use of ruxolitinib solely for its survival benefit, that is, in patients without splenomegaly or troublesome symptoms, as it appears that the survival benefit may be indirect, via increases in appetite, weight, energy level and overall feeling of well-being. Conversely, we do use it in symptomatic, low or intermediate-1 risk patients, in line with guidelines issued by the US National Comprehensive Cancer Network.^9▪^ We base the starting dose of ruxolitinib on the platelet count and, in general, try to optimize the dose as spleen responses to ruxolitinib are dose-dependent and correlate with survival.^10^ Anemia and thrombocytopenia are on-target, dose-limiting toxicities of ruxolitinib. However, ruxolitinib-induced anemia does not carry the adverse prognosis of disease-associated anemia and, ruxolitinib, in fact, may overcome the negative prognostic impact of the latter.^11^,^12^ We attempt to counteract ruxolitinib-induced cytopenias, most severe during the first 12 to 24 weeks, with the use of erythroid stimulating agents, danazol or low dose thalidomide^13^ and transfusion support, so as to avoid dose reductions and interruptions of ruxolitinib treatment during this critical early period during which spleen response is greatest. The activin receptor ligand traps have shown promise in the treatment of anemia of myelofibrosis, both as monotherapy and in patients receiving ruxolitinib,^14^ and may soon become available for anemic patients with lower risk myelodysplastic syndromes. Vaccination against shingles using the inactivated virus (i.e., not live attenuated) is recommended in patients receiving ruxolitinib. We occasionally use splenectomy for patients with symptomatic splenomegaly that is refractory to drug therapy, and often associated with hypersplenism. For patients proceeding to allo-HCT, we advocate performing the procedure around the time of best response to ruxolitinib, and continuing ruxolitinib up until initiation of the conditioning regimen. For patients with prefibrotic PMF, we generally recommend observation, as the data on interferon^15^,^16^ require further validation in our opinion, and discontinuation rates can be high because of adverse effects, while managing bleeding/thrombotic risk as is done for ET.^17^

## Future perspectives: unmet needs and investigational approaches

While the optimal definition of “ruxolitinib failure” is debatable, the median duration of spleen response to ruxolitinib is about 3 years.^6▪^^,^^7▪^ Preclinically, “type 1 JAK2 inhibitor persistence”^18▪^ has been shown to perhaps explain the development of clinical resistance to ruxolitinib, and can be reversed by temporarily withdrawing the drug. Indeed, there have been anecdotal reports of restoration of clinical responsiveness to ruxolitinib upon re-challenge.^19^ However, this is an area of significant unmet need. Patients who discontinue ruxolitinib have a poor outcome, and clonal evolution and worsening platelet counts while on ruxolitinib predict for worse survival upon discontinuation.^20^ The investigational JAK2 inhibitors, pacritinib and fedratinib, and the JAK1/2 inhibitor, momelotinib, have demonstrated some efficacy in the postruxolitinib setting, and regulatory approval of one or more of these agents would be a very welcome development.^21^ Additionally, being nonmyelosuppressive, pacritinib could possibly fill the therapeutic void for patients with platelets <50 × 10^9^/L, while momelotinib may improve anemia, possibly through activin receptor antagonism.^22^

Many therapeutic avenues beyond JAK inhibition have been explored in clinical trials (see Table ^1^ for a listing of selected recently reported monotherapy and combination approaches). Interesting survival data were recently released for the telomerase inhibitor, imetelstat, in ruxolitinib-exposed patients.^23^ While the median survival of 29.9 months in the higher dose (9.4 mg/kg) arm is certainly impressive, a “real world” study from Italy reported a median survival of 22.6 months among 171 patients discontinuing ruxolitinib,^24^ substantially higher than that reported by 2 large US academic centers.^20^,^25^ The very nontoxic anti-fibrotic compound, recombinant pentraxin-2, PRM-151, yielded bone marrow reticulin and collagen fibrosis improvements in approximately half the patients in a small (n = 18) open-label extension study, generally corresponding to improvements in cytopenias.^26^ This agent has been studied both alone and in combination with ruxolitinib, and data from a fully accrued study of 3 different doses of this drug in ruxolitinib-pretreated patients are eagerly awaited. While a number of frontline ruxolitinib-based combination strategies have been disappointing, encouraging results have been reported for the combinations with azacitidine,^27^ sotatercept,^14^ and thalidomide.^13^ Other combination trials have taken an “add-on” approach, where an investigational agent is added to ruxolitinib in patients having a suboptimal response to ruxolitinib monotherapy.^28^,^29^ A multitude of laboratory-based, synergistic or otherwise logical combination strategies, as well as novel single-agent approaches exists, some already in the clinic and others awaiting translation; these have recently been reviewed by the authors.^21^ The search for the holy grail of truly disease-modifying drugs or drug combinations continues.

**Table 1:**
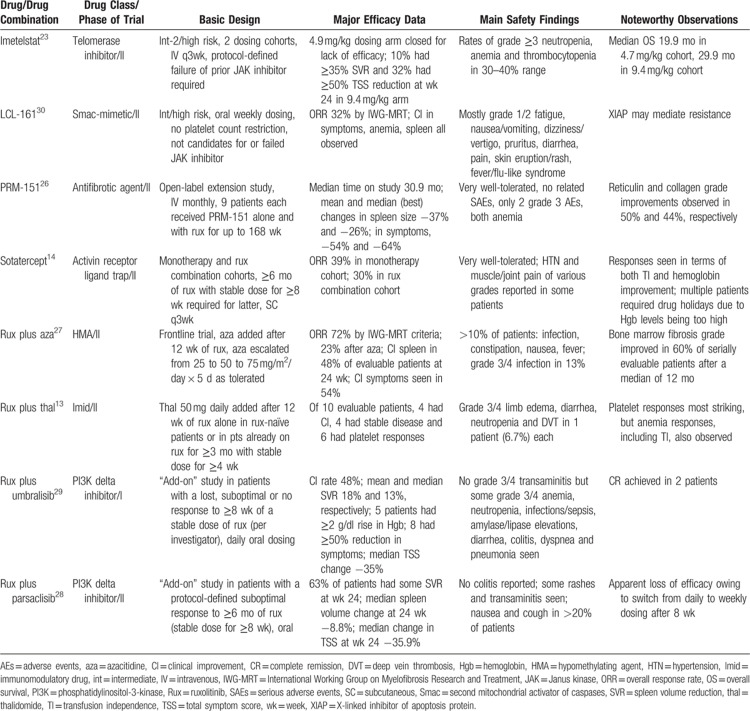
Selected Clinical Trials of Novel Agents and Combinations in Patients With Myelofibrosis
